# 3D angle-independent Doppler and speckle tracking for the myocardium and blood flow

**DOI:** 10.1530/ERP-19-0040

**Published:** 2019-09-27

**Authors:** Norman McDicken, Adrian Thomson, Audrey White, Iqbal Toor, Gillian Gray, Carmel Moran, Robin J Watson, Tom Anderson

**Affiliations:** 1 Centre for Cardiovascular Research, School of Clinical Sciences and Community Health, The University of Edinburgh, Edinburgh, UK

**Keywords:** ultrasound, Doppler, speckle tracking, ratio indices, 3D, angle-independence

## Abstract

A technology based on velocity ratio indices is described for application in the myocardium. Angle-independent Doppler indices, such as the pulsatility index, which employ velocity ratios, can be measured even if the ultrasound beam vector at the moving target and the motion vector are not in a known plane. The unknown plane situation is often encountered when an ultrasound beam interrogates sites in the myocardium. The velocities employed in an index calculation must be close to the same or opposite directions. The Doppler velocity ratio indices are independent of angle in 3D space as are ratio indices based on 1D strain and 1D speckle tracking. Angle-independent results with spectral Doppler methods are discussed. Possible future imaging techniques based on velocity ratios are presented. By using indices that involve ratios, several other sources of error cancel in addition to that of angular dependence for example errors due to less than optimum gain settings and beam distortion. This makes the indices reliable as research or clinical tools. Ratio techniques can be readily implemented with current commercial blood flow pulsed wave duplex Doppler equipment or with pulsed wave tissue Doppler equipment. In 70 patients where the quality of the real-time B-mode looked suitable for the Doppler velocity ratio technique, there was only one case where clear spectra could not be obtained for both the LV wall and the septum. A reproducibility study of spectra from the septum of the heart shows a 12% difference in velocity ratios in the repeat measurements.

## Introduction

The diversity of Doppler ultrasound technology in medicine continues to increase (1, 2). This paper summarises developed Doppler technology which is usually beam/velocity angle dependent. It then describes the use of velocity ratio indices which are angle independent in 3D. Techniques developed in one field, for example, blood flow, can readily be translated to another for example, tissue motion. The Doppler effect is the basis for very sensitive tools for the detection of myocardial motion and blood flow.

This paper aims to present useful features of velocity ratio techniques with particular emphasis on myocardial motion.

The backscattered ultrasound signal from blood can originate from overlapping echoes from blood cells or groups of cells (rouleaux). For myocardial tissue the dominant signal is from small structures and gives rise to a coarse speckle pattern in the B-mode image unlike the fine speckle pattern seen in a B-mode image of blood. The exact small structures are usually not known but they can still be used as markers for myocardial motion. Doppler signals can be measured at specific sites and times. Doppler techniques are fast and also very sensitive since they use frequencies which can be measured accurately and can also be filtered from background noise. A limitation of current Doppler techniques, which measure velocity, is that they are dependent on the angle between the ultrasound beam and the velocity directions. This means that the sonographer must always be mindful of the angle at which Doppler measurements are taken. This paper also discusses briefly strain ratio indices which can readily be derived and which are independent of beam and velocity directions in 3D. Strain is of interest in the myocardium since it can be related directly to function.

### Doppler-based indices

**Figure 1:**
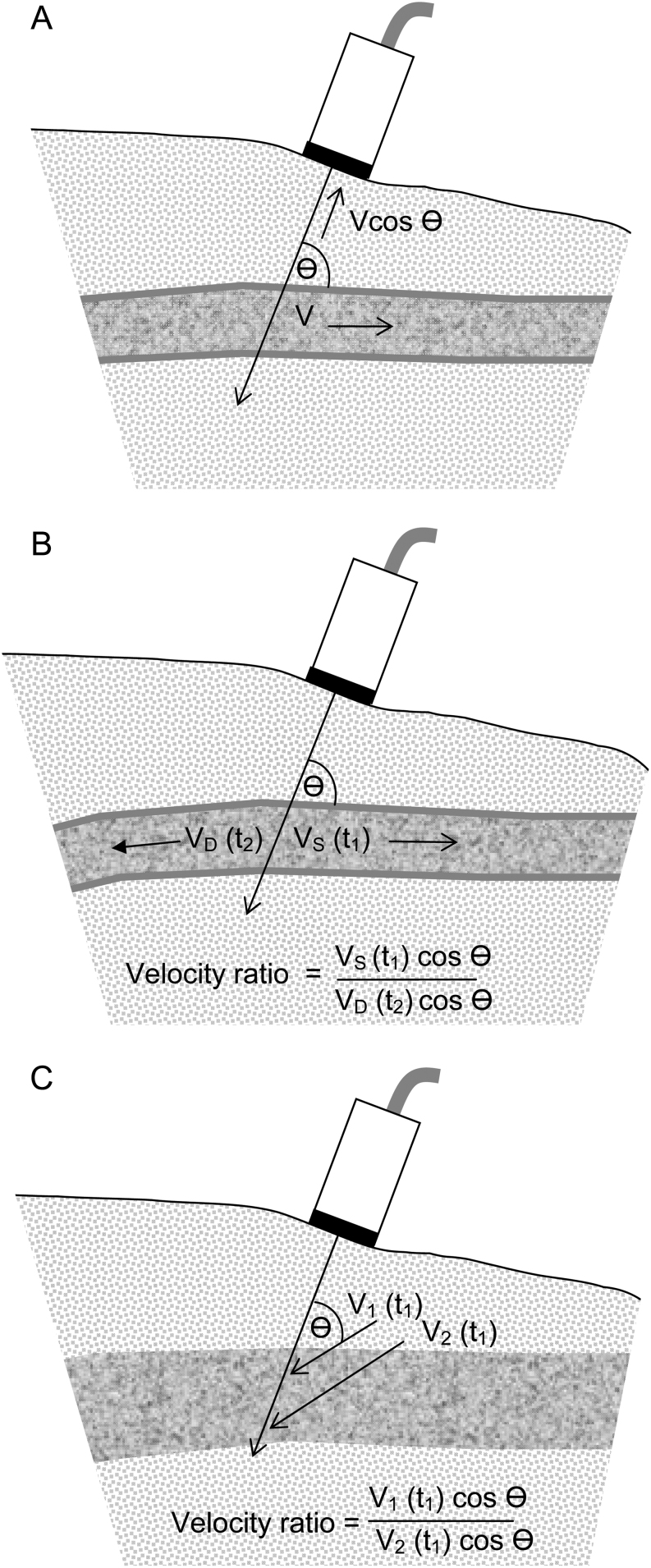
Ultrasound beam intersecting motion at angle θ. Moving tissue and beam in a 2D plane. (A) Velocity component Vcosθ. (B) Velocity ratio for tissue motions in systole (S) and diastole (D). (C) Tissue velocities measured at neighbouring depths for calculation of velocity ratio.

Indices, for example pulsatility index (PI) and resistance index (RI), have been defined and extensively evaluated for blood flow and the status of blood vessels (3, 4, 5). Measurement of these indices is comparatively simple and is of value in clinical practice. Theses indices employ ratios of velocities and hence cancel the unknown angle factor (Fig. 1A, B and C). Quantitative indices provide means of classifying stage in disease development. For the present technique, two indices are of particular interest, the velocity ratio index (VRI) and the velocity gradient ratio index (VGRI). VRI is the ratio of velocities at two neighbouring locations or times, say v1/v2. VGRI is the ratio of velocity gradients at two neighbouring locations, say (v1 − v2) / (v2 − v3). Velocity difference is often called velocity gradient in cardiology.

The situation when taking ratios is often envisaged in a 2D plane; however, ratio cancellation is valid in 3D. In other words the ultrasound beam may approach the moving target from any direction in 3D and still provide a correct result. When the direction of a beam intersecting a moving target is changed in 3D space, a new plane is defined in which the beam and velocity intersect at an angle. VRI should be of value in assessing myocardial activity which is often in an unknown direction in 3D. This 3D angle-independent aspect of ratio indices has not been reported previously for cardiac tissue motion or blood flow possibly because blood vessels and flow are often envisaged or imaged in 2D and the out-of-plane direction is ignored.

The calculation of strain rate and strain in the myocardium with current Doppler techniques uses velocities and are therefore angle dependent. Strain rate and strain ratio indices are independent of angle in 3D. All of the above ratio indices should be applicable with transthorasic, transesophageal and catheter scanning where beam/angle manipulation is restricted.

**Figure 2:**
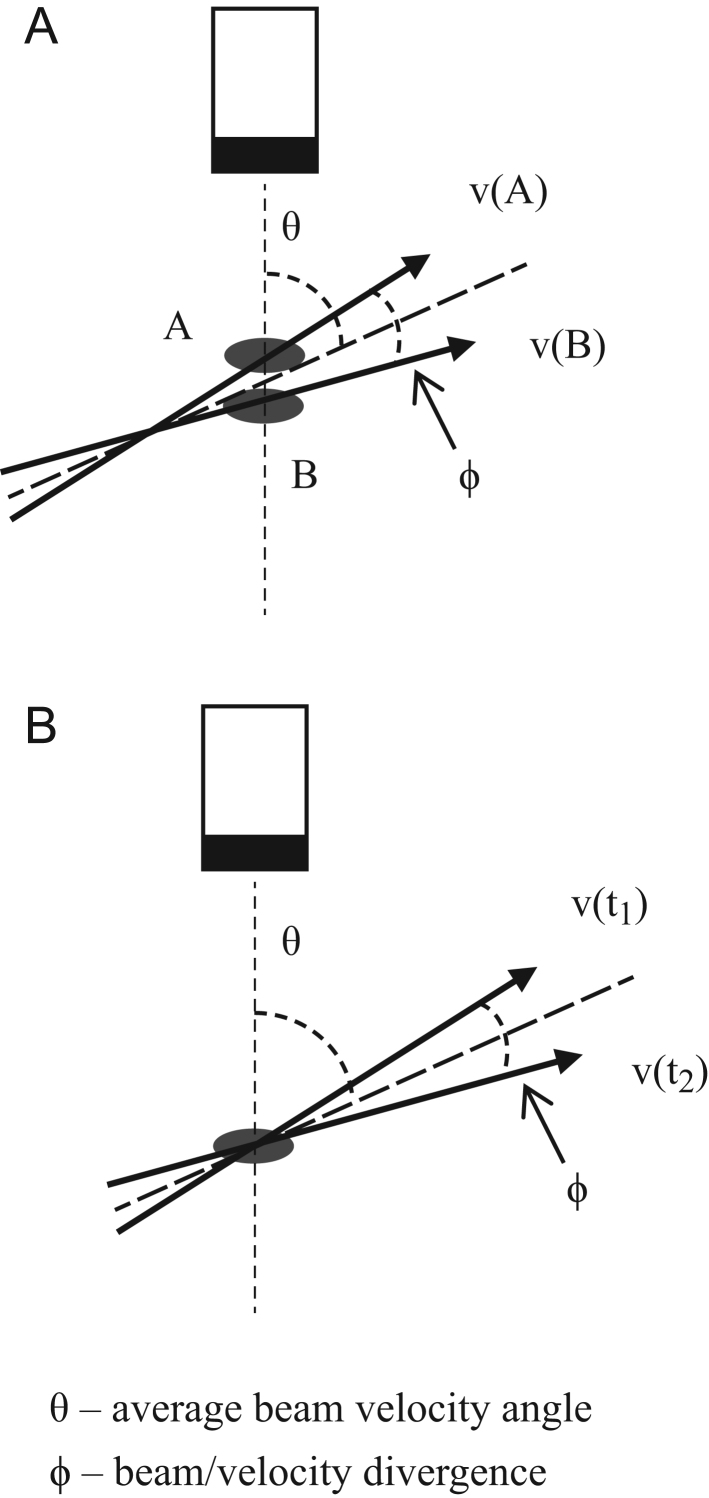
Beam velocity angles. (A) Beam velocity angle θ. (B) Velocity divergence angle φ.

Velocities employed in ratios are required to be close to the same (0°) or opposite (180°) directions. Some difference in angle from the angles of 0° or 180° is tolerated. When a beam interacts with two targets moving with the same speed at an insonation direction of 0° but diverging from each other by 5° the difference in the velocity components measured is 1% (Fig. 2). At an insonation direction close to 20° the difference is 3%, only above 50° is the difference greater than 10%. At an insonation direction above 60°, the difference rapidly rises so higher angles are to be avoided. The Doppler indices are therefore not sensitive to beam/velocity angle and are usable at many sites in the myocardium. The insonation angle can be checked with real-time B-mode or velocity vector speckle tracking (6). For neighbouring tissues these velocity vectors are often surprisingly parallel even when there is twisting of the ventricle. Scrutiny of 30 ultrasound B-mode and MRI images support this observation (7, 8).

### Doppler technology

A number of Doppler technologies have been developed. The following list presents the more common ones which could be combined with the velocity ratio technique.

1. Continuous wave Doppler blood flow spectroscopy,
2. Pulsed wave Doppler blood flow spectroscopy,
3. Power Doppler,
4. Duplex real-time B-mode/Doppler blood flow spectroscopy,
5. Colour flow imaging (CFI),
6. Doppler tissue spectroscopy,
7. Doppler tissue imaging,
8. Duplex real-time B-mode/CFI,
9. Real-time velocity vector imaging,
10. Ultrafast Doppler imaging,
11. 3D colour Doppler imaging,
12. Transophageal Doppler,
13. Catheter Doppler.

The aim of this paper is to demonstrate by discussion of published data and by experimental measurement that indices can readily be produced which are related to velocities within the myocardium and which are angle independent in 3D.

## Developments in myocardial velocity measurement

### Velocity generation in myocardium

**Figure 3:**
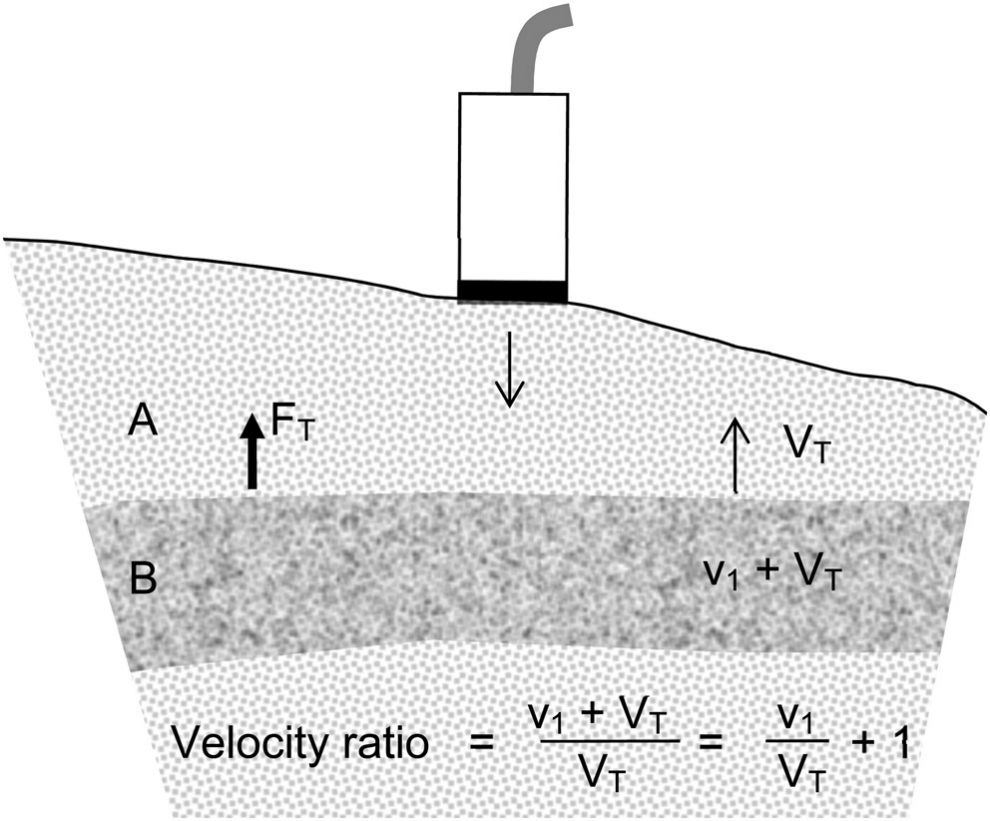
Layer B tethered to layer A. Force F_T_ generated by A acts on B. Velocity ratio for velocities in A (V_T_) and in B (v_1_ + V_T_). The internally generated velocity in B is v_1_.

When considering the velocities at specific locations in the myocardium, two effects produce the velocities. One, v_1_, is due to the local contraction or relaxation within the muscle, the other, V_T_, is due to the motion of adjacent tissues linked to the site of interest that is the tethering effect (Fig. 3). In our study of the mouse posterior LV wall, typical values of v_1_ and V_T_ were found to be 0.15 and 2 cm/s respectively at peak diastole. Since tethering velocity affects the numerator and denominator in VRI in a similar way, this index is insensitive to tethering. When V_T_ changes from 2 to 6 cm/s, VRI changes by 11%.

Since the effect of tethering is difficult to assess in practice, experimental and clinical studies are required to support this insensitivity conclusion. VGRI eliminates the effect of the tethering velocity by the subtraction process inherent in its calculation. Using the mouse posterior LV wall data, VGRI was calculated to be 1.3. More data are required than could be collected with the present system (Philips IE33 scanner incorporating a Doppler Tissue Imaging mode) to permit usable accuracy in the measurement of VGRI. VGRI requires additional measurements of velocity to enable the velocity gradients to be obtained. Also the subtraction process adds error. A system that allows this process to be carried out quickly would be best suited to the calculation of VGRI. Since VGRI is valuable in that it is independent of tethering such systems could be developed in future. VGRI is not considered further in this study.

### Doppler tissue developments

Imaging of myocardial velocities and measurement of velocity gradient, that is, the difference of velocities in neighbouring tissues, have been successfully applied in research (9, 10, 11, 12). Velocity gradient is equivalent to strain rate and is angle dependent which may limit its application as a clinical tool. On the other hand, velocity gradient ratios (strain rate ratios) are angle independent in 3D. Ultrafast Doppler imaging systems have been described for imaging of tissue motion and blood flow in the heart (13, 14). Frame rates up to 20,000 per second have been achieved in 2D and 3D. More parameters such as mean or peak velocity and bandwidth are available at each pixel for imaging and calculation of strain. The small footprint transducers required to access the heart result in angle-dependent Doppler velocity components being imaged therefore ratio indices should be useful. In some artery scanning, linear arrays can be employed thereby permitting a number of beam directions at each sample volume and hence two or three velocity components can be combined to give angle-independent velocity vectors in 2D (15).

Doppler spectra gathered at specific sample volumes (pixels) have been applied to assess pathological conditions in the myocardium (16). Blood vessel wall motion can be imaged and stiffness studied (17). Velocity gradient has been applied to calculate strain in the myocardium (18). Doppler images of tissue acceleration have been employed to determine the intramural site of the origin of myocardial contraction in response to electrical stimulation (19). With current Doppler techniques, knowledge of the angle of incidence should be ascertained if possible so that small angles can be used where the dependence of the velocity measurement on angle is low. Alternatively it may be possible to compare measurements from normal and abnormal tissue states even when the Doppler angle is above 30° where the cosθ factor in the Doppler velocity formula starts to change quickly with angle.

### Doppler strain indices

The calculation of strain rate and strain in the myocardium with current Doppler techniques uses velocities and are therefore angle dependent. Strain rate and strain ratio indices are independent of angle in 3D.

### Image speckle tracking developments

A high temporal resolution sequence of 1D speckle echo traces from a fixed direction pulsed beam through the myocardium can be used to study tissue motion (20). The measured velocity component of motion along the ultrasound beam is angle dependent. As for Doppler, ratios of these speckle velocity components are angle independent in 3D. 1D speckle ratios have not been used to date in research or clinical application.

Growing interest in imaging velocities in the myocardium led to the application of 2D image speckle tracking in this field in an attempt to produce results which are not dependent on an unknown angle of incidence of the ultrasound beam at the site of interest. Since cardiac motion occurs in 3D space there is interest in developing speckle tracking techniques which measure or allow for 3D motion. Research and commercial development of 2D and 3D tracking of the echo speckle pattern in images for blood flow and tissue motion have been pursued for a number of years (21, 22). This subject has been reviewed (23). Speckle tracking of myocardial tissue has been successful though limitations due to beam degradation and some angle dependence are still to be evaluated. Some angle dependence has been noted since speckle patterns can be tracked more accurately for motion along the ultrasound beam than across it (24, 25). Considerable effort is being made to develop 2D and 3D image speckle tracking. The latter aims to cater for the 3D motion of the myocardium and hence avoid through-plane artefacts which can be present in 2D imaging. Errors due to noise and also ease of clinical application are also being assessed. Three-dimensional speckle tracking has been reviewed (26). At present errors in 2D speckle strain measurement are typically 20–30%. Errors in 3D speckle tracking are larger due to the slower frame rates.

### Other sources of error

In addition to angle-dependent errors, other sources of error may be reduced or eliminated by using ratio indices. Less than optimum scanner control settings that govern sensitivity are often used and this affects measurements such as peak or mean velocity. In particular, it is usually not possible to avoid errors in depth gain compensation when imaging dynamic blood filled cavities. Errors due to beam degradation factors, which affect sample volume shapes, are also reduced that is errors due to refraction, attenuation and non-linear propagation. Ultrasound beams are often substantially distorted as they propagate through tissue. Errors due to focusing and transducer size should also be reduced. Errors in electronics and computer algorithms affect the numerator and denominator velocity values in the ratio indices and hence cancel. This cancellation of errors means that discrepancies obtained with machines from different manufacturers should also be reduced. Such discrepancies can be problematic when results from different centres are being compared. Since the velocities used in the ratios will be affected in a similar manner by loading, the ratio technique is expected to be independent of loading though this still requires to be tested. The benefits of employing ratios are similar to those found in audible acoustics when decibel measures, which employ ratios of acoustic pressure, are used to cancel unknown errors due to microphones, amplifiers, filters and so forth.

### Application of velocity ratio and strain ratio indices

In myocardial tissue applications, three ways have been identified where VRI may be used:

1. Measurement of velocities in neighbouring tissues for example at locations across or along a myocardial wall in humans and mice. Observation of ultrasound images of the myocardium of neighbouring tissues reveal that they move in similar directions at each instant (Fig. 4).

**Figure 4:**
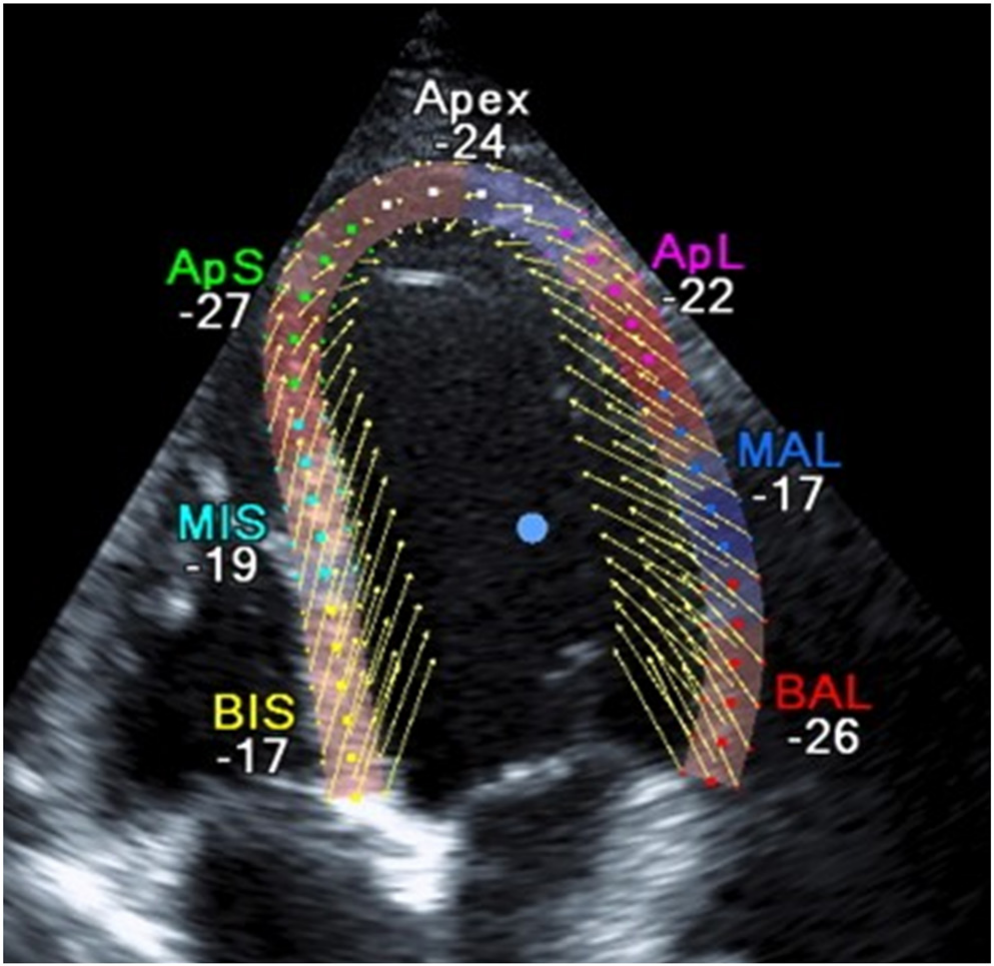
Velocity vectors derived by speckle tracking. Neighbouring tissues move in similar directions.
2. Measurement of the velocity of a particular structure at different times in the cardiac cycle for example peak systolic velocity and peak diastolic velocity. Observation of real-time ultrasound B-mode images of particular structures and MRI tissue tracking reveal that they often retrace their paths at similar angles in systole and diastole (Fig. 5). The cosine factors have the same magnitude for each direction and therefore cancel.

**Figure 5:**
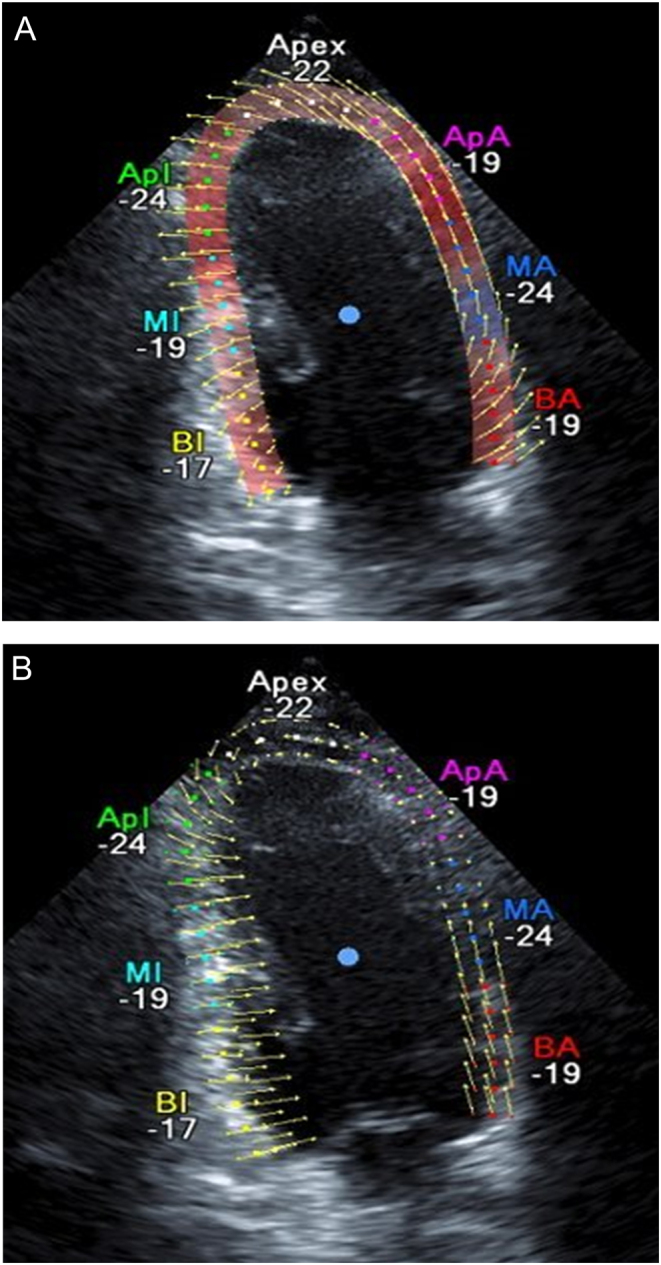
Velocity vectors derived by speckle tracking. Reversed tissue motion during diastole (A) and systole (B).
3. Measurement of velocities at a particular site over time such as when they change in response to a drug such as Dobutamine in stress testing (Fig. 6).

**Figure 6:**
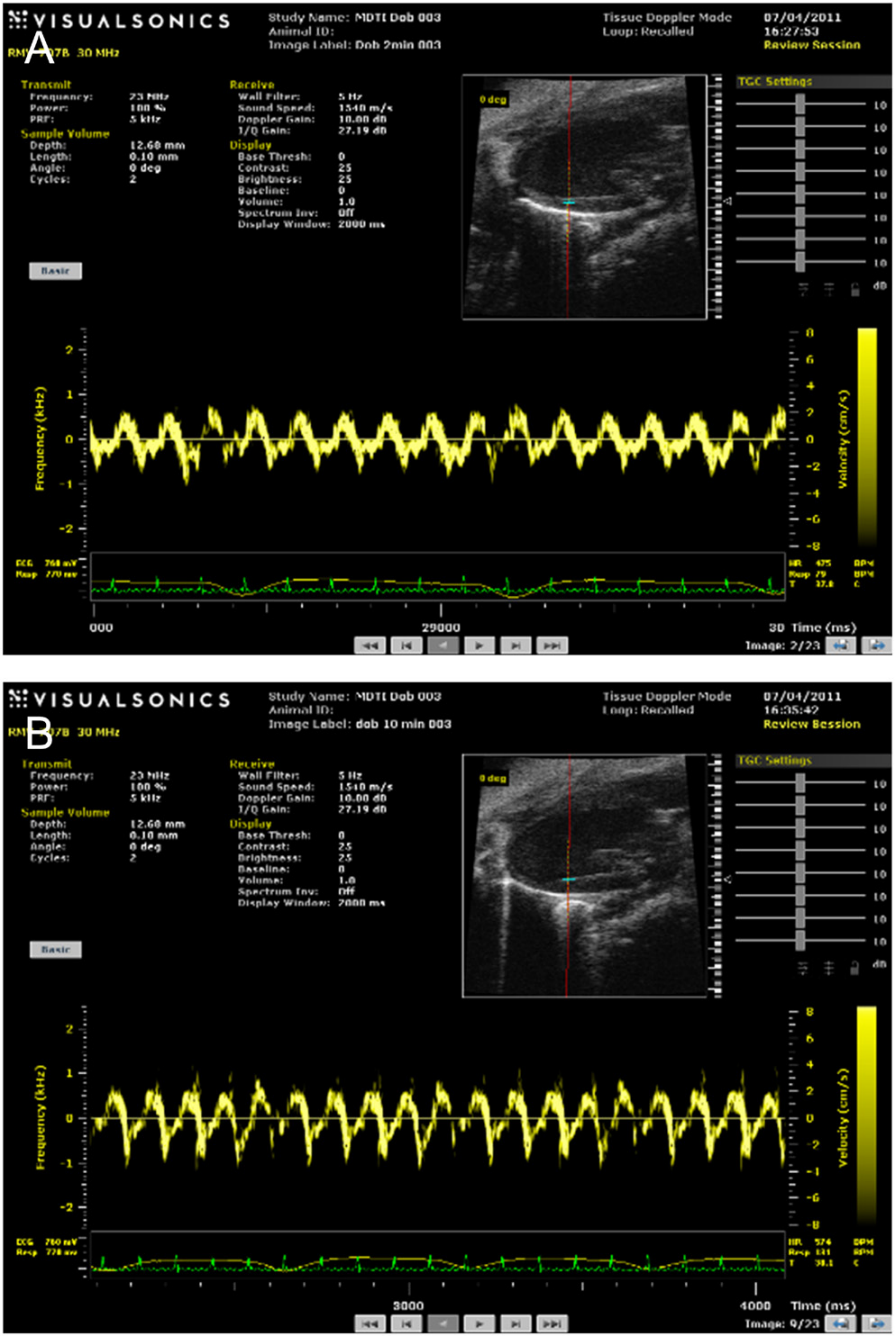
Spectra from left ventricle wall of a mouse at (A) 2 min and (B) 10 min after injection of Dobutamine. VRI for peak diastolic velocities (10 min/2 min) is 1.4. Cancellation of angle factor occurs if geometry held fixed.

Velocity ratios for neighbouring sites across a blood vessel or at the same site at different times will also be angle independent in three dimensions if the velocities are in the same or opposite direction.

## Methods and results

The aim of this section is to demonstrate that in practice:

1. The velocity patterns in the myocardium are often as required for the calculation of ratio indices.
2. Ultrasound data can readily be obtained for such calculations.

Three commercially available scanners were employed in this study of myocardial motion with velocity ratios: a FUJIFILM VisualSonics Inc. (Toronto, Canada) scanner (VEVO 770) with a mechanically oscillating transducer RMV 707B operating at 30 MHz for mice and two Philips Healthcare phased array scanners (IE33 and Affiniti 70C) with the same transducer operating at 3.4 MHz for humans. The VisualSonics permitted grey shade real-time B-mode and grey-shade M-mode as well as Pulsed Doppler Spectral recording. The Philips machines offered grey-shade real-time B-mode, grey-shade M-mode, Colour Doppler Tissue real-time B-mode (DTI), Colour Doppler Tissue M-mode, Pulsed Doppler Spectrorgram recording and Speckle Vector Velocity imaging.

The Visualsonics VEVO Pulsed Doppler mode was employed to scan mice. The mice were placed on a heated operating table in the supine position and their paws were attached to *in situ* electrodes to monitor ECG and respiration. Temperature was monitored via a rectal thermometer and stabilised to 37 ± 0.5 °C. Hair was removed from the mouse chest with a chemical hair remover to minimise ultrasound attenuation. The mice were anaesthetised with a 2% isoflurane/oxygen mixture. Anaesthetisation was confirmed with responsiveness to toe pinches. Aquasonic 100 gel was applied between the skin and the transducer. The technique was developed using 30 mice, no recordings were discarded.

The VRI, calculated from the spectra, has proven to be suitable for probing the myocardium. For example Fig. 6 shows spectra from which the derived VRI (peak diastolic velocity/peak systolic velocity) is not subject to angle errors in 3D in studies of the response to Dobutamine. Similar spectra were obtained for 70 patients with one technical failure. The strong echoes from tissue resulted in clearly defined maximum velocities in the spectra. A reproducibility study from the cardiac septum showed a 12% difference in velocity ratios in the repeat measurements.

## Doppler velocity ratio imaging (DVRI) and M-mode (DVRM)

**Figure 7:**
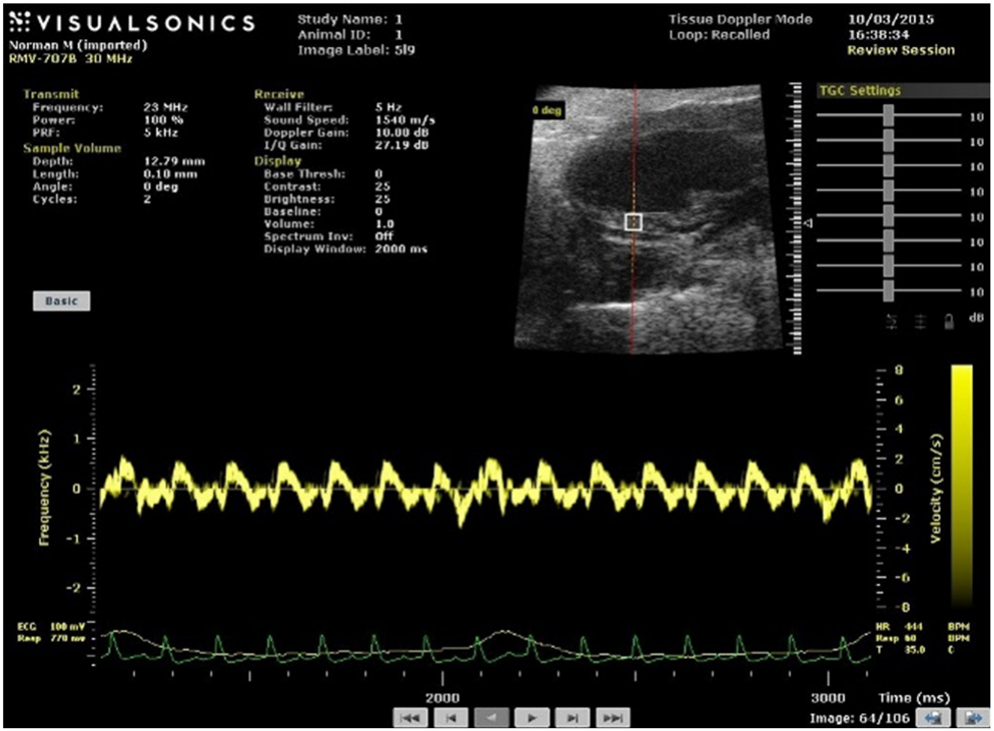
Array of pixels located on selected mouse myocardial region. Sample volume dimensions at each pixel are 0.1 x 0.1 mm. Typical spectrogram from left ventricle wall. Peak diastolic/peak systolic velocity ratio (VRI) is 0.8.

To investigate DVRI, a 10 by 10 array of tissue Doppler sample volumes was superimposed on the B-mode image of the left ventricle lateral wall of a mouse (Fig. 7). A sample volume of length 0.1 mm was systematically moved through the area of the box generating a 10 × 10 array of velocity data. A spectrogram was obtained for each sample volume (pixel) to provide data for an image of the myocardium in the array. In the present study, spectrograms of the mouse myocardium were obtained for a 10 by 10 array of sample volumes each of 0.1 by 0.1 mm.

The parameters extracted from each spectrogram were the peak diastolic and peak systolic velocity. VRI were then calculated for locations in the myocardium which were to be compared.

**Figure 8:**
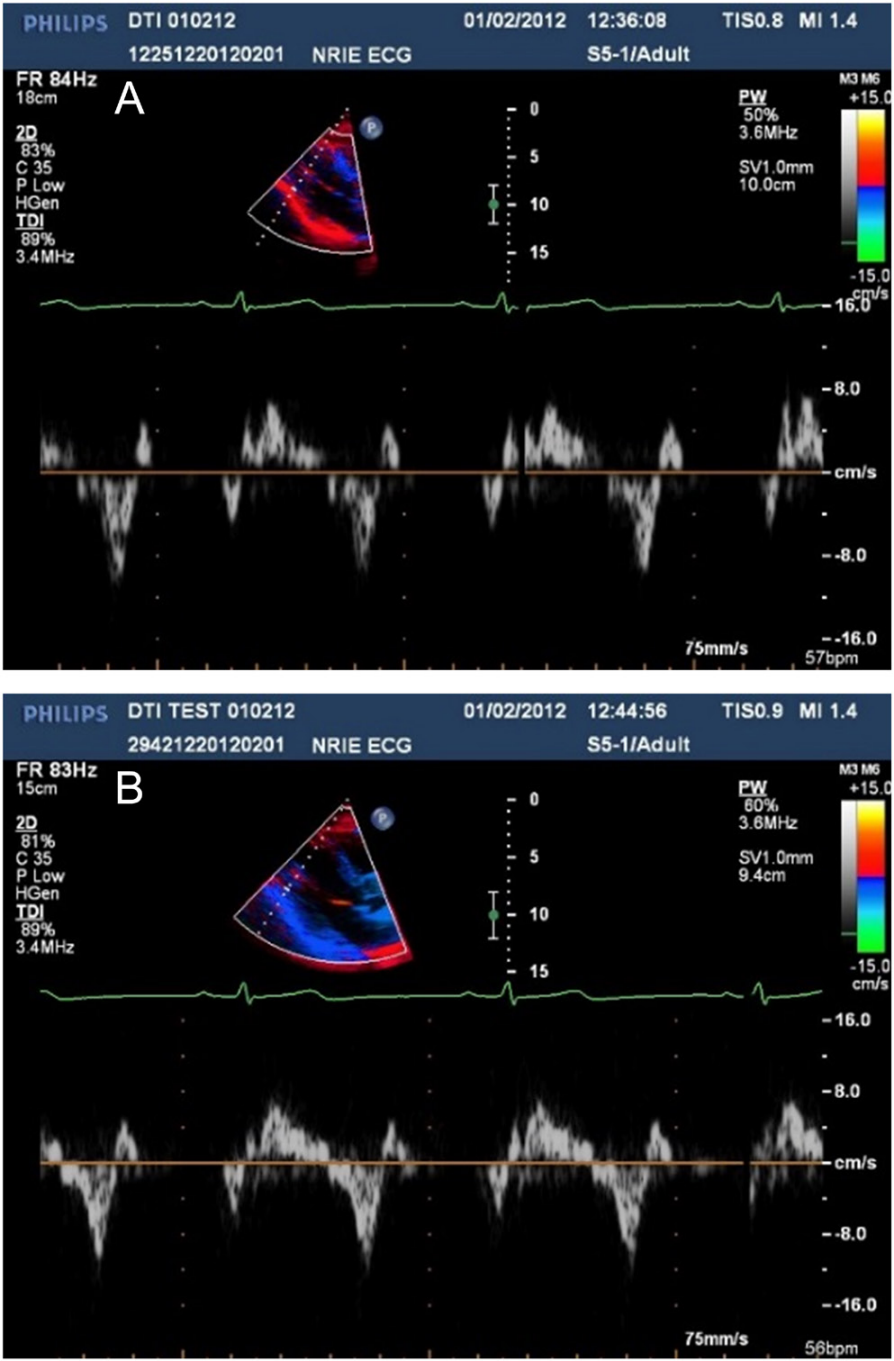
Spectrogram from pixels located in normal human left ventricular wall. Depths of sample volumes 9.4 cm and 10.0 cm. Sample volume length 1.0 mm. VRI for peak diastolic velocities is 1.2.

**Figure 9:**
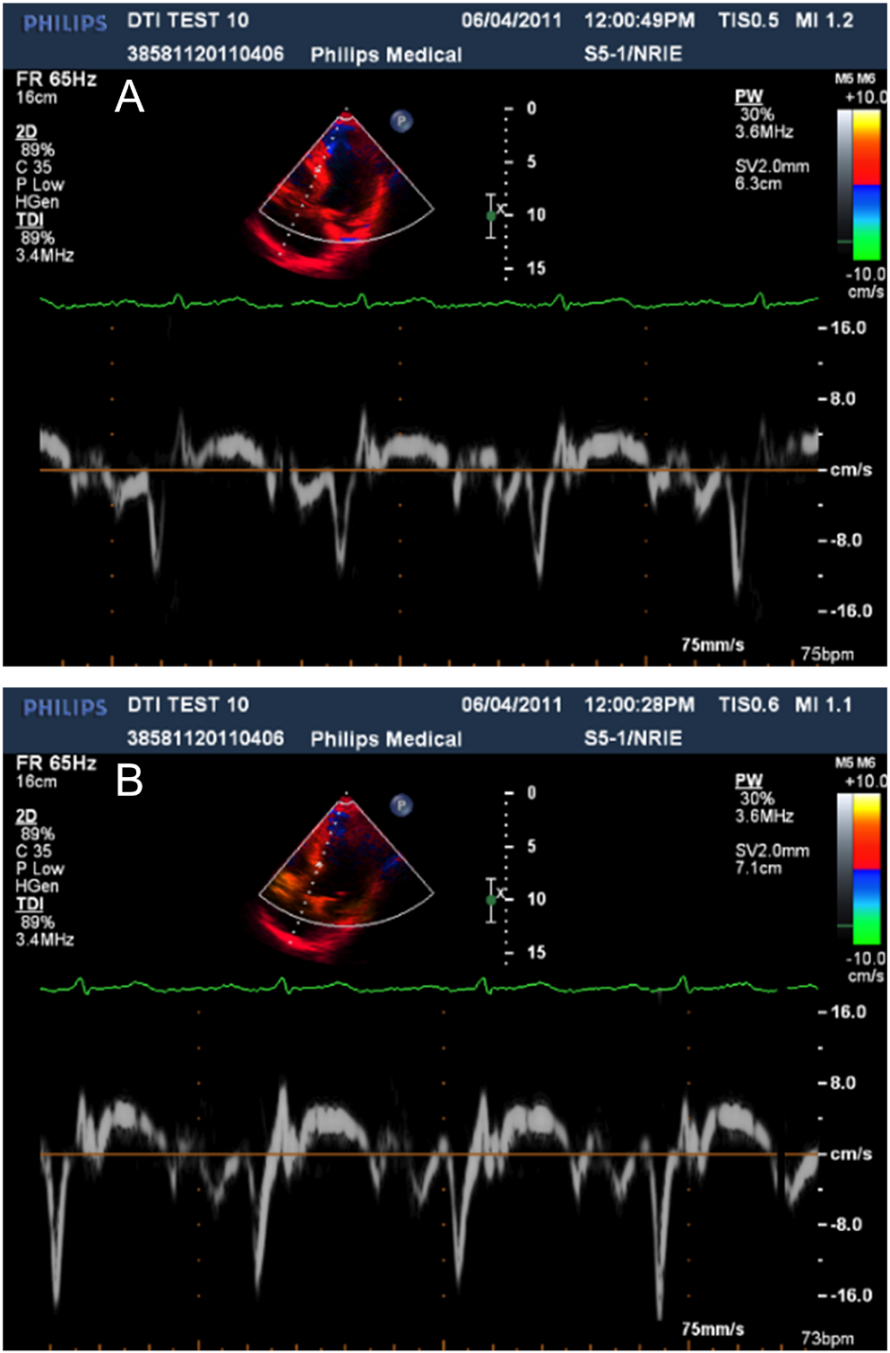
Spectrogram from pixels located in normal human left ventricular septum. Depths of sample volumes 6.3 cm and 7.1 cm. Sample volume length 1.0 mm. VRI for peak diastolic velocities is 1.6.

In the human heart studies, one line of sample volumes, each of length 1.0 mm, straddled the posterior wall of the left ventricle (Fig. 8) and interventricular septum (Fig. 9). The acquisition time, for example, 5 min, for spectra in a 2D array of sample volumes with the available system was acceptable for research but not clinical application. Mean velocity at each pixel could have been acquired using the conventional DTI images but for clarity of interpretation, peak velocity was preferred. For these reasons human studies were carried out with a single line of spectral Doppler sample volumes in the myocardium.

**Figure 10:**
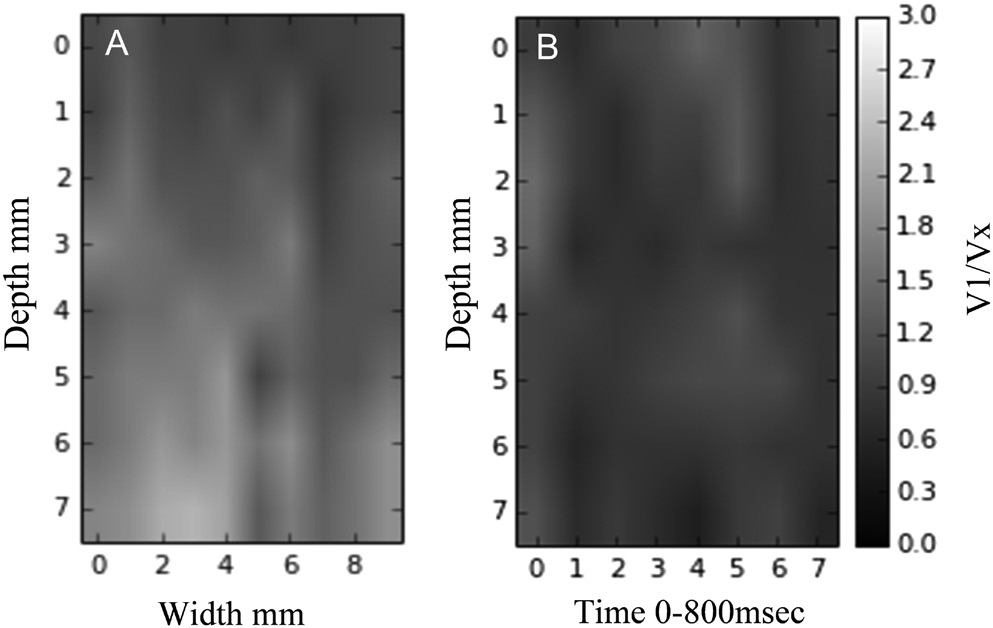
(A) B-mode of velocity ratio indices. (B) M-mode of velocity ratio indices (grey shades denote ratio of anterior velocity over velocity at depth).

Two-dimensional mouse images of the VRI were produced to explore the feasibility of creating images which are angle independent in 3D (Fig. 10A). The ultrasound beam was stepped across the field-of-view. Images of VGRI require a greater collection of data than is possible by the present data collection approach. With more data, averaging techniques should then reduce errors arising in the calculation of VGRI.

With the beam held in a fixed direction, M-mode velocity ratio images, analogous to M-mode DTI, are also possible (Doppler velocity ratio M-mode, Fig. 10B). They have the attraction of presenting 3D angle-independent motion information using a single beam direction.

The human research was carried out in accordance with the policies of the Lothian Ethical Committee, Scotland. Each patient gave informed consent for spectra to be collected from 4 or 6 sites in the myocardium. The data collected was anonymised.

Small animal experiments were approved by the University of Edinburgh Animal Welfare Review Board and by the Home Office.

## Discussion

Doppler techniques are sensitive, fast and noise resistant. This is particularly true of spectral and M-mode application. Most research, aimed at measuring motion in 3D, exploits speckle tracking. The relatively slow speed of 3D scanning presents challenges. The Doppler ratio approach may provide simple, robust and fast techniques of value in some applications.

At angles of incidence near 90° the velocity ratio approach is still valid but the Doppler shifts are small and approach zero. In this situation it is usually possible to approach the site of interest from a different angle. It may be possible in the future to employ ratios of velocity bandwidths which are usable at all angles and for which sample volume distortion effects cancel (27, 28).

Ideally Doppler information would be extracted simultaneously whether from a linear array or a two-dimensional array of sample volume pixels. The sensitivity and accuracy of the velocity ratio techniques remain to be evaluated.

**Figure 11:**
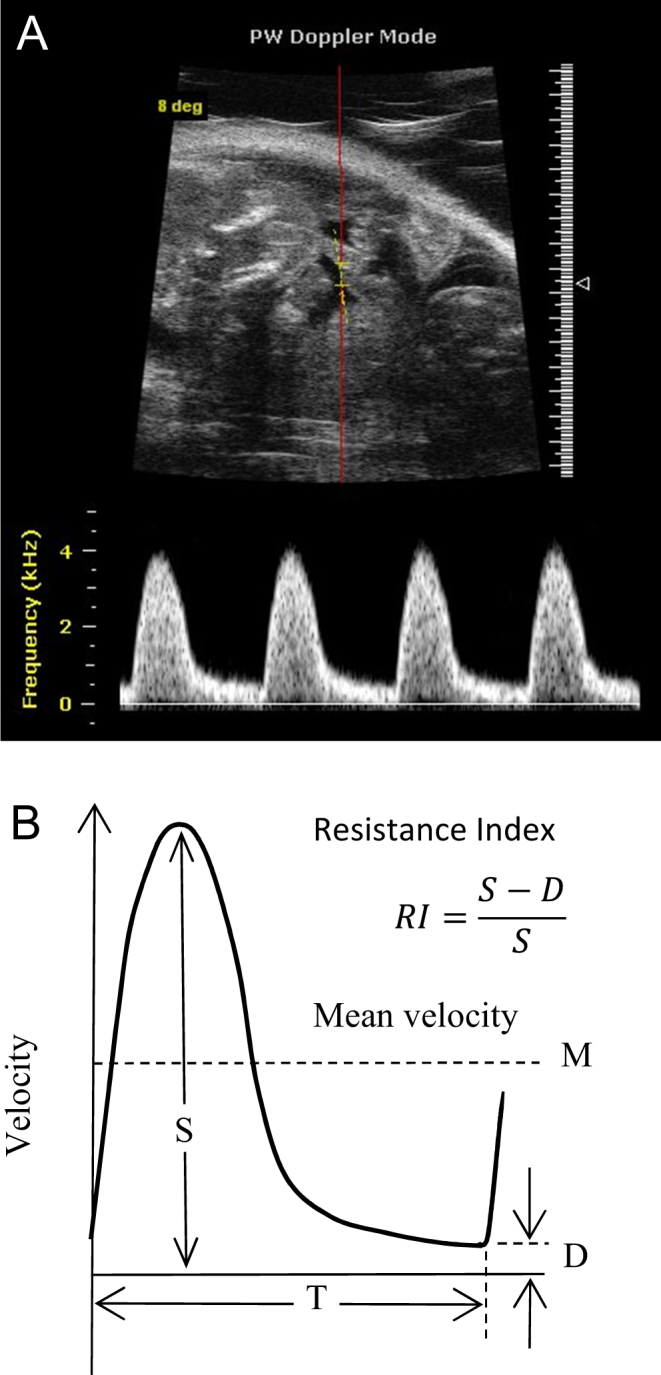
A Doppler signal from a mouse umbilical artery for which the angle between the ultrasound beam and the flow velocity vector is not known in 3D. In this case the pulsatility index (PI) is 2.1.

Most of the discussion in this paper relates to tissue in the myocardium. However, the ratio indices are valid for blood flow where the angle between the ultrasound beam and the blood velocity vector is not known in 3D. Figure 11 illustrates the measurement of the RI for the case of blood flow in a mouse umbilical artery for which the beam/velocity angle is not known in 3D.

### Future work and conclusions

This work demonstrates that in practice Doppler velocity data from the myocardium can be used to generate indices that are angle independent in 3D. These indices may be derived from spectral Doppler, M-mode tissue Doppler and B-mode tissue Doppler. B-mode and M-mode type images of velocity ratios can be produced. Ratios of strain rate and strain are angle independent in 3D and will be explored further. Further work will be undertaken to explore the accuracy and clinical value of Doppler ratio methods. The present system is simple but widely available and could be expanded using known imaging technology.

This paper introduces the concept of VRI being used to study tissue motion and blood flow which do not occur in a known plane. A hybrid approach will be investigated in which 2D speckle velocity Imaging will be used to ascertain that the Doppler angle is not close to 90° and that the velocities used in the ratios are close to being in the same or exactly opposite directions. Errors in Doppler index techniques will be evaluated with known motion in phantoms and *in vivo*. Other errors deriving from machine characteristics or ultrasound propagation will also be studied. The influence of physiological parameters such as loading remains to be investigated. It may well be that loading affects the measurement of velocities in a similar manner and hence the effects of loading cancel in ratios. Clinical studies are required to evaluate VRI in practice. The insensitivity of ratios to errors arising from the technology should help to produce a robust clinical tool.

## Declaration of interest

The authors declare that there is no conflict of interest that could be perceived as prejudicing the impartiality of the research reported.

## Funding

Resources and scientific support were provided by the Pre-Clinical Research Centre of the University of Edinburgh. Grant funding was provided by the Welcome Trust (UK) Grant WT 083327A1A.
